# Time-resolved neutron scattering provides new insight into protein substrate processing by a AAA+ unfoldase

**DOI:** 10.1038/srep40948

**Published:** 2017-01-19

**Authors:** Ziad Ibrahim, Anne Martel, Martine Moulin, Henry S. Kim, Michael Härtlein, Bruno Franzetti, Frank Gabel

**Affiliations:** 1 Université Grenoble Alpes, Institut de Biologie Structurale, 38044 Grenoble, France; 2 Centre National de la Recherche Scientifique, Institut de Biologie Structurale, 38044 Grenoble, France; 3 Centre à l’Energie Atomique et aux Energies Alternatives, Institut de Biologie Structurale, 38044 Grenoble, France; 4 Institut Laue-Langevin, 38042 Grenoble, France

## Abstract

We present a combination of small-angle neutron scattering, deuterium labelling and contrast variation, temperature activation and fluorescence spectroscopy as a novel approach to obtain time-resolved, structural data individually from macromolecular complexes and their substrates during active biochemical reactions. The approach allowed us to monitor the mechanical unfolding of a green fluorescent protein model substrate by the archaeal AAA+ PAN unfoldase on the sub-minute time scale. Concomitant with the unfolding of its substrate, the PAN complex underwent an energy-dependent transition from a relaxed to a contracted conformation, followed by a slower expansion to its initial state at the end of the reaction. The results support a model in which AAA ATPases unfold their substrates in a reversible power stroke mechanism involving several subunits and demonstrate the general utility of this time-resolved approach for studying the structural molecular kinetics of multiple protein remodelling complexes and their substrates on the sub-minute time scale.

AAA+ (ATPases Associated with diverse cellular Activities) unfoldase complexes represent a large family of protein remodelling machines which play an essential role in the clearance of damaged or misfolded proteins as well as in specific regulatory tasks, including the degradation of cell-cycle components, transcription factors and metabolic enzymes^1^,^2^,^3^. The degradation of most proteins in cells is catalysed by large proteolytic complexes that hydrolyse ATP and proteins through linked reactions^4^,^5^,^6^. Energy-dependent proteases are found across the three domains of life: bacteria contain multiple AAA+ proteases (ClpXP, ClpAP, HslUV, FtsH and Mpa:20S), archaea have two known cytoplasmic AAA+ proteases (PAN:20S and Cdc48:20S) and, in eukaryotes, a large part of cytosolic proteins are degraded by the 19S:20S system forming the 26S proteasome^2^. In these systems the AAA+ proteins form a hexameric ring complex that associates with the catalytic core particles. The common feature believed to underlie the diverse functions of the AAA+ family of ATPases is their ability to undergo structural alterations during the ATP power stroke that cause unfolding of proteins or disassembly of protein complexes^2^,^7^. In the case of proteolytical AAA ATPases, ATP-binding and -hydrolysis fuel changes in the conformation of the rings which cause movements of the axial pore loops implicated in substrate translocation and unfolding^8^,^9^. Despite the central importance of the nucleotide-dependent, coordinated conformational changes in AAA+ proteolytical unfoldases, and while progress has been obtained recently on the sequential order of nucleotide binding and hydrolysis^10^, little is known on the potential coordination of these events with the time course of substrate unfolding and release^11^.

AAA+ unfoldase activities have been mostly studied by following the loss of fluorescence of proteins such as green fluorescent protein (GFP)^12^. Other biophysical methods, including single-molecule optical trapping^13^ or Förster resonance energy transfer (FRET)^14^ have been applied to monitor unfolding by AAA+ enzymes. While these techniques provide accurate kinetic data and working models, direct structural information on AAA+ unfoldases or their protein substrates during the unfolding reaction cannot be extracted straightforwardly from the data. On the other hand, high (and low) resolution structural studies, carried out on different AAA+ proteasome regulators^15^,^16^,^17^, often represent static snapshots of one (or a few) conformations of the unfoldase complexes in the presence or absence of nucleotide. A central unsolved challenge in dissecting the mechanisms of these complicated machines is therefore to determine how ATP-binding and -hydrolysis coordinates the conformational changes that ultimately allow substrate unfolding. Although different models have been proposed in literature^1^,^11^,^18^, the paucity of time-resolved biophysical and structural methods to test specific models has limited the insight into these machines. In particular, many experimental techniques lack the ability to provide structural information on AAA+ proteases during their unfolding activity and on the time course of protein substrate structural changes and the state in which they are released.

Small-angle neutron scattering (SANS), combined with solvent contrast variation (H_2_O/D_2_O ratio) and specific deuteration of proteins, is a particularly well-suited technique to monitor and separate conformational changes of individual partners within a complex by highlighting or suppressing their respective signals^19^,^20^,^21^. Basic, model-free information are radii of gyration and molecular masses of visible partners (i.e. molecules having a scattering contrast) in solution, averaged over exposure time and all conformational sub-states. More sophisticated information are low-resolution shapes and pseudo-atomic models if complementary structural data of subunits (e.g. from NMR or crystallography) are available^22^. For weakly scattering biological samples such as dilute (~mg/ml) protein solutions, SANS is usually used in “static” mode with exposure times of several minutes up to hours per sample. In order to obtain time-resolved structural data, we coupled short SANS frames (30 seconds) at a high flux neutron diffractometer (D22 at Institut Laue-Langevin Grenoble, France) with a specifically developed online fluorescence device to record data from solutions of alternately perdeuterated (i.e. 100% deuterated) and reconstituted unfoldase-substrate mixtures. This novel combination of time-resolved SANS, combined with online fluorescence and temperature activation, allowed us to monitor and dissect the structural kinetics of the unfolding process of a tagged GFP substrate (GFPssrA) from the concomitant conformational changes of the archaeal PAN (proteasome activating nucleotidase) unfoldase. Importantly, by using the hyperthermophilic PAN complex from *Methanocaldococcus jannaschii*, the unfolding reaction could be controlled and slowed down by working at 55–60 °C (i.e. lower than the physiological conditions), and allowed us to extract and dissect the respective structural kinetics of PAN and GFP during the active unfolding process.

Our data support a molecular mechanism in which ATP hydrolysis results in a large conformational contraction of the PAN complex at 55–60 °C, providing a reversible power stroke and a force needed to unfold GFP. The contraction is followed by a relaxation of the PAN complex to recover its initial conformation after complete unfolding of GFP and can be described by a bi-exponential function with characteristic times of a few hundred seconds. The protein substrate is progressively unfolded and released within 1–2 minutes in a (partially) unstructured, destabilized state in solution and subsequently forms specific aggregates under our experimental conditions *in vitro*. Interestingly, the “peristaltic pumping motion”^23^ or “power stroke” of the PAN ensemble occurs on a similar time scale as the unfolding (half-life ~7 minutes) and translocation of the GFP substrates.

These findings provide a new basis to explain several functional and mechanistic aspects of the PAN complex and other AAA+ unfoldase machineries and stress the need for a tight interplay and coordination of AAA+ unfoldase and peptidase partners and their assembly into functional complexes in order to avoid uncontrolled aggregation of unfolded/destabilized protein substrates *in vivo*. Finally, the novel combination of time-resolved SANS, online spectroscopy and the use of temperature-activatable, hyperthermophilic archaeal systems appears to be very promising to study a wide variety of macromolecular complexes (and their substrates) involved in protein remodelling and proteome homeostasis.

## Results

### Purification and biophysical characterization of a functional hexameric PAN complex from *Methanocaldococcus jannaschii* and its interaction with GFP substrates

**Figure 1:**
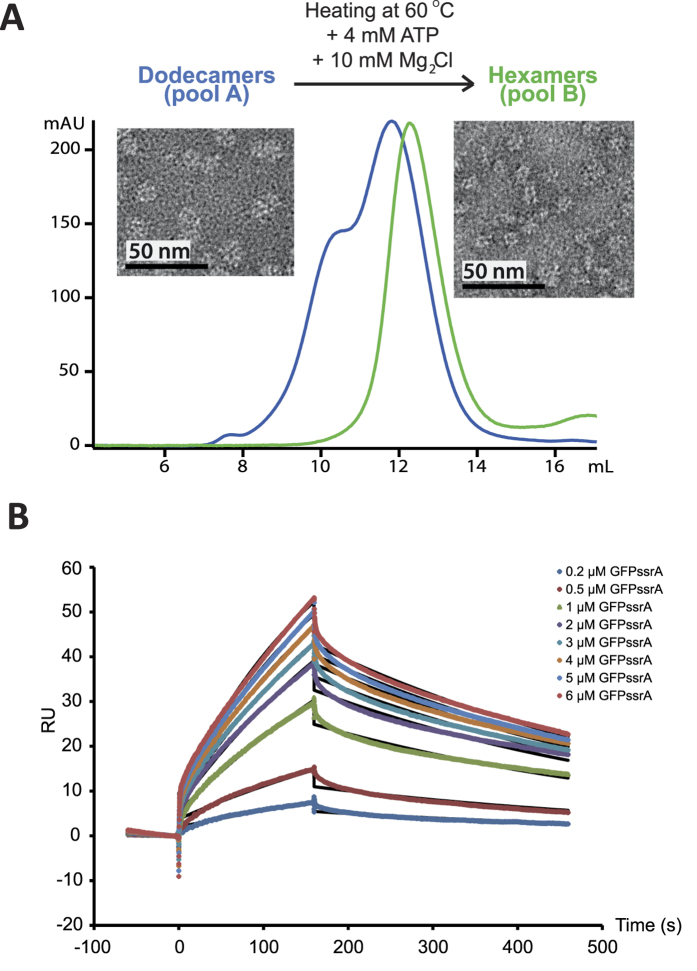
Purification and biophysical characterization of the *Mj*PAN complex. (**A**) Superose 6 column chromatography purification profiles of the dodecameric/hexameric mixture (blue chromatogram) and the hexameric (green chromatogram) forms of *Mj*PAN complexes. Negative stain transmission electron micrographs of the *Mj*PAN complex in its dodecameric (pool A) and hexameric (pool B) forms are shown next to the corresponding chromatogram. (**B**) Kinetic analysis of the interactions of PAN with GFPssrA. The colored lines represent binding responses for injections of protein analyte at specified concentrations (μM) over the PAN-coated surface. The kinetic data were fitted (black curves) by a 1:1 Langmuir binding model^45^ which describes monovalent analyte binding to a single site on the immobilized ligand (see SI Material and Methods).

Sample quality of biomacromolecules is crucial for structural interpretation of solution scattering data^24^ and we characterized the PAN-GFP system by several biophysical techniques prior to the SANS experiments. A preliminary gel filtration analysis showed that the purified PAN complexes were assembled as a mixture of dodecamers and hexamers (Fig. 1A, blue curve). By heating the dodecamers at 60 °C in the presence of ATP and MgCl_2_, it was possible to isolate monodisperse hexameric *Mj*PAN particles that remained stable up to high concentration (**~**20 mg/ml) (Fig. 1A, green curve). In addition, the gel filtration column fractions were analysed by negative stain electron microscopy (Fig. 1A) and Size Exclusion Chromatography, coupled with Small Angle X-ray Scattering (SEC-SAXS) (Fig. S1). The two peaks from the Superose 6 gel filtration column (see Methods) corresponded to the dodecameric and the hexameric *Mj*PAN, confirming the isolation of a monodisperse solution of the *Mj*PAN hexamers after heating with ATP. Finally, the oligomeric state of PAN was validated directly during the SANS experiments by the scattered intensities in the forward direction (Eq. 1), *I*(0), and was in good agreement with the theoretical values predicted for a hexamer (Supplementary Table S1). Together, these results highlight the important role played by ATP hydrolysis and high temperature for the generation of a monodisperse, hexameric PAN solution.

The kinetics and affinity of the interaction were measured by Surface Plasmon Resonance (SPR) prior to SANS experiments. The sensorgram (Fig. 1B) shows the binding of a range of concentrations of GFPssrA when injected across the PAN-coated sensor-chip surface in the presence of 2 mM ATP at 45 °C. The association rate constant (*k*_a_), dissociation rate constant (*k*_d_) and overall equilibrium dissociation constant (*K*_D_) were approximated by a 1:1 Langmuir binding model (SI Material and Methods) which allowed to determine the association (*k*_a_ = 512.3 M^−1^.s^−1^) and dissociation (*k*_d_ = 0.0022 s^−1^) rate constants as well as the affinity (*K*_D_ = 4.28 μM) of the *Mj*PAN:GFPssrA interaction. In conclusion, GFPssrA bound to PAN specifically with a low μM affinity, characterized by a very slow on-rate (association) followed by a slow off-rate (dissociation) as expected in the presence of an ssrA tag^25^.

### GFP is unfolded by PAN and forms specific aggregates on the time scale of minutes

**Figure 2:**
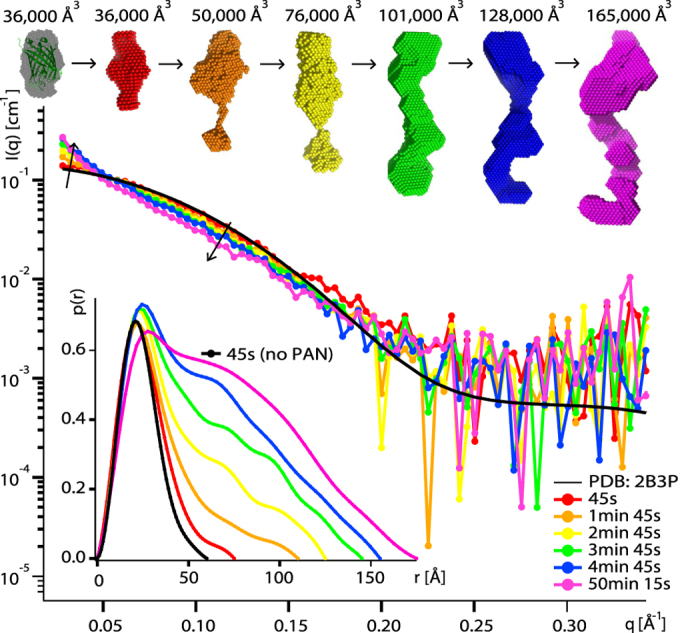
GFPssrA is released and aggregates in solution after unfolding by PAN. Middle: SANS curves recorded on deuterated (d) dGFPssrA (2 mg/ml) in the presence of hydrogenated (h) hPAN (10 mg/ml) and ATP (100 mM) in a 42% D_2_O buffer (hPAN invisible) at 55 °C during the unfolding reaction. The back-calculated SANS curve (CRYSON^35^) from the GFP crystal structure (PDB ID 2B3P) is shown as a black continuous line. Top: *ab initio* envelopes of dGFPssrA at different times and the respective volumes of each model. The first model (grey) is generated from SANS data without hPAN (control experiment, see Fig. S3) and is superimposed with the crystal structure. Bottom left: pair distance distribution functions, P(r), calculated using GNOM^46^.

**Figure 3:**
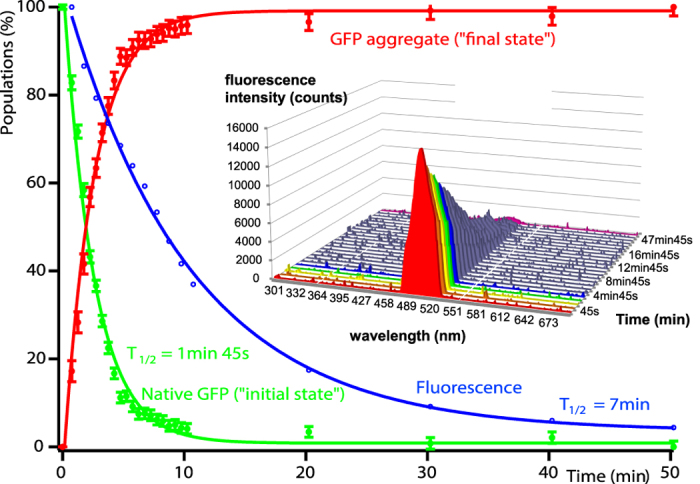
Time-resolved quantification of native and aggregated GFPssrA populations during unfolding by PAN. Curves showing the populations of natively folded GFPssrA (green) and its aggregates (red) during the unfolding reaction. Continuous lines display fits with Eq. 2. Inset: UV fluorescence spectroscopy emission spectra of GFP in the presence of PAN and ATP at 55 °C recorded on the same samples and at the same time as the SANS measurements. The GFP emission peak fluorescence at 509 nm is plotted in blue and was fitted by a single exponential decay function (Supplementary Eq. 4).

The kinetic structural information obtained by time-resolved SANS, in combination with online fluorescence spectroscopy (Supplementary Fig. S7), allowed us to follow the unfolding of the GFPssrA substrates by PAN *in vitro* on the time scale of minutes (Figs 2 and 3). Working with perdeuterated GFP molecules and hydrogenated (“natural”) PAN molecules allowed masking the SANS signal of the latter in 42%/58% D_2_O/H_2_O solutions used (Supplementary Fig. S2)^21^. SANS profiles of GFPssrA were evolving continuously over time in 30 s intervals as the protein was being unfolded at 55 °C (Fig. 2) while in a control experiment in the absence of PAN, the scattering profiles of isolated GFPssrA remained virtually unchanged for almost an hour showing only slight modifications toward the end, probably due to a minor thermal denaturation and/or dimerization resulting from the prolonged exposure at 55 °C (Supplementary Fig. S3). Concomitantly, unfolding resulted in an increase in substrate aggregates, accompanied by a loss of the fluorescence signal (Fig. 3). Within 45–75 s, GFP started to being unfolded and losing its native, globular structure through intermediate, elongated structural states before its release in solution and subsequent aggregation (Fig. 2). After 1 min 45 s, first GFP aggregates appeared as seen through the scattering curves and the generated *ab initio* models.

With increasing time, both SANS and fluorescence data show that the unfolded GFP molecules, once released by PAN, were losing their native structure and consequently their solubility, and interacted to form larger aggregates. Indeed, a continuous increase in zero angle scattering *I*(0) (Supplementary Table S1), volumes of the *ab initio* models (Fig. 2), as well as the respective populations determined by the OLIGOMER fit (Fig. 3) illustrate the build-up of larger aggregates with time. It is interesting to note, however, that the model extracted from the first scattering curve (recorded after ~45 s) yielded the same volume as the one generated from the control sample in the absence of PAN (native GFP), but displayed more elongated shapes (Fig. 2, red model). Thus, after 45 s of the reaction, contamination with aggregates appears to be minor but a significant population of the GFP molecules are unfolded (or partially unfolded), giving rise to the elongated shape envelope of the protein and the increase of the maximum dimension D_max_ of the respective pair distance distribution functions P(r). Using a simplified two-state model (Eq. 2), it was possible to describe the GFP populations during unfolding by two species as a function of time; the initial folded (native) GFP and the aggregates after 50 min (Fig. 3). While after ~10 min most of isolated native GFP (green curve) was lost and clustered into large aggregates (red curve), the decrease of fluorescence (blue curve) was slower and it took more time (~50 min) for complete disappearance suggesting that some aggregated GFP molecules were still fluorescing.

Several control conditions (use of non**-**hydrolysable ATP analogue ATPγS and absence of nucleotides) were probed at different temperatures in order to assess the role of ATP in the unfolding process. The results show that ATPγS blocks GFPssrA unfolding by PAN since the SANS profiles remained identical over 50 min at 55 °C (Supplementary Fig. S4). Similarly, when the experiment was carried out in the absence of nucleotides, no structural changes in GFP were observed. However, in both cases a small decrease in the emission fluorescence at 509 nm (Supplementary Fig. S4) suggested a weak local destabilization of GFPssrA without large structural alterations or aggregate formation since the SANS curves remained stable and superimposable throughout the reaction. These findings indicate that, even in the absence of a hydrolysable nucleotide, PAN and its protein substrate interact *via* the ssrA tag.

In summary, the time-resolved SANS approach, coupled to online fluorescence, provided structural data that confirm that PAN releases destabilized and unfolded substrates in solution within minutes (in the experimental conditions applied here) when it functions alone and without the involvement of other cellular partners like the 20S catalytic particles or other components such as molecular chaperones. The unfolding process of protein substrates seems to be processed in a unidirectional way allowing the formation of long, (partially) unfolded substrates (15–20 Å longer than the native folded GFP) as intermediate states before the accumulation into larger aggregates. Interestingly, a comparison with SANS control data obtained by forced thermal denaturation of GFP (Supplementary Fig. S5) yielded slightly different types of SANS curves and P(r) functions indicating that the underlying molecular mechanisms of protein destabilization by PAN and by heat, respectively, produce distinct unfolded states with different propensities and modes of aggregation.

### An ATP-induced, reversible conformational contraction of PAN occurs during substrate unfolding

**Figure 4:**
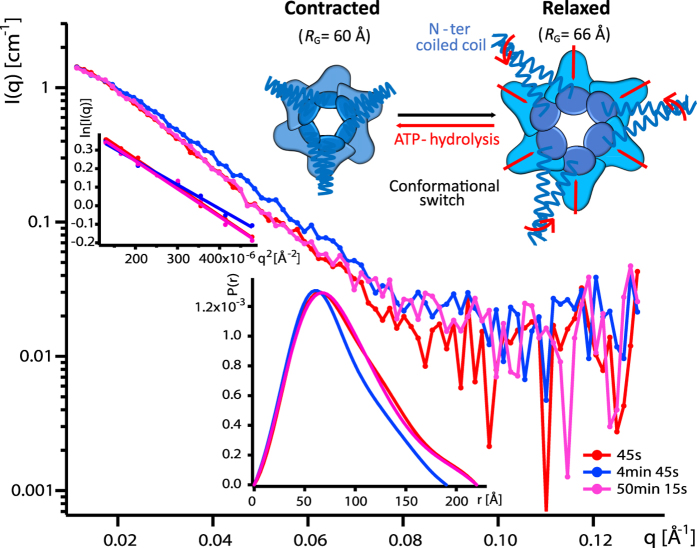
ATP-induced reversible contraction of PAN during substrate unfolding. Middle: Scattering curves recorded on deuterated (d) dPAN (3 mg/ml) in the presence of hydrogenated (h) hGFPssrA (4.8 mg/ml) (invisible in 42% D_2_O buffer) at 60 °C with 30 s exposure time per curve during different times of the unfolding process. Inset, left and bottom: Guinier fits and pair distance distribution functions, P(r), calculated using GNOM^46^. Inset, top: schematic representation of a putative conformational transition from a relaxed to a contracted PAN state upon ATP hydrolysis involving N-ter coiled coil movements and C-ter ATPase domain conformational changes, rotations and translational movements.

**Figure 5:**
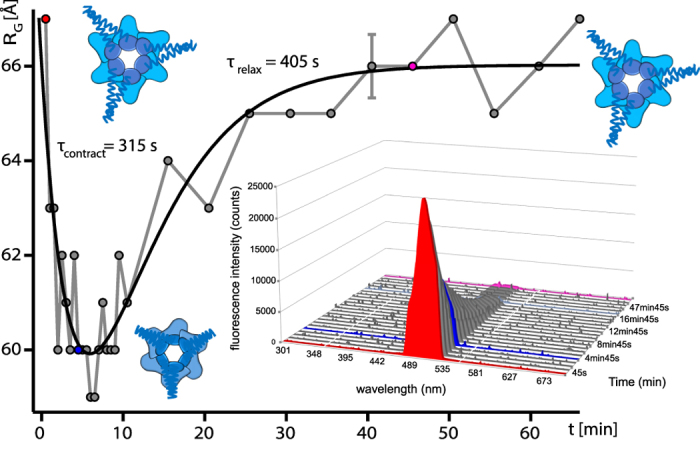
Time-dependence of the contraction and re-expansion of PAN during substrate unfolding. Evolution of the (ensemble-averaged) PAN radius of gyration as a function of reaction time. PAN is shown schematically in a contracted and extended state. A two exponential function (Eq. 3) is fitted against the experimental data (black curve). Inset: UV fluorescence spectroscopy emission spectrums of GFPssrA in the presence of PAN and ATP at 60 °C recorded on the same samples and at the same time as the SANS measurements. A single, typical experimental error bar is shown for the *R*_G_ data at 40 min.

In analogy to GFP, the kinetics of PAN conformational changes were monitored by time-resolved SANS with 30 second time resolution at 60 °C in the presence of ATP and GFPssrA substrate, coupled with online fluorescence spectroscopy (Supplementary Fig. S7). In order to focus on the signal of PAN and mask the signal of GFP, a reverse labelling scheme was applied, i.e. perdeuterated PAN and hydrogenated (“natural”) GFP were reconstituted in a 42%/58% D_2_O/H_2_O buffer (Supplementary Fig. S2). The SANS profiles of PAN showed a clear evolution within several minutes during the unfolding process toward a more compact form with a diminished maximum extension (Fig. 4, blue curve). Indeed, both Guinier analysis and P(r) after 4 min 45 s showed a maximum decrease of ~6 Å in the radius of gyration and more than 20 Å decrease in D_max_ with respect to the initial state after 45 s (Figs 4 and 5). Forward scattered intensities *I*(0) remained relatively stable in the same time range (Supplementary Table S1), indicating that the oligomeric state of PAN remained intact and that the *R*_G_ differences corresponded to conformational changes within the hexamer. Interestingly, toward the end of the unfolding reaction, PAN adopted again its initial conformation. Indeed, the scattering curves recorded after 50 min superimpose nicely over the whole *q*-range with the first curve (45 s). Likewise, the Guinier analysis of the first and final curves yielded the same radius of gyration (~66 Å, Fig. 5) and the maximum dimension (D_max_) of the molecule at the end of the reaction calculated from the P(r) function was identical to the one at the beginning (Fig. 4, inset).

The contraction/relaxation mechanisms of PAN during substrate unfolding could be adequately described by a bi-exponential time function (Eq. 3, Methods) of the experimental radius of gyration *R*_G_ (Fig. 5, black curve). The characteristic time parameters τ_contract_ and τ_relax_, describing the time course of PAN contraction and relaxation during substrate unfolding were 315 ± 25 and 405 ± 30 s, respectively. At the end of the reaction (~50 min) the fluorescence signal was negligible (Fig. 5, inset), the whole GFPssrA substrate was unfolded, and PAN was relaxed and re-expanded to recover its initial, non-contracted conformation. Further control experiments, without nucleotide or with ATPγS, in the presence of GFPssrA, were performed at 60 °C and showed that PAN, in the absence of a hydrolysable nucleotide, did not undergo conformational changes under these conditions (Supplementary Fig. S6).

## Discussion

### Time-resolved SANS coupled to fluorescence: a novel approach to study the structural and functional kinetics of proteome remodelling machines

Previous biophysical studies measured PAN, ClpA or ClpX unfolding of GFPssrA by changes in native fluorescence^12^,^25^, single molecule optical trapping^26^,^27^ and Förster resonance energy transfer (FRET)^14^. However, to the best of our knowledge, none of these techniques have been used to determine the evolution of structural parameters of both substrates and AAA+ enzymes as a function of time during an active unfolding process. The time-resolved SANS approach presented here, coupled to fluorescence and combined with temperature activation, has several advantages with respect to more “classical” approaches like crystallography and electron microscopy that yield static snapshots of single or a few isolated “trapped” states. Due to the high performance of the D22 diffractometer at the Institut Laue-Langevin neutron facility, combined with perdeuteration, it was possible to run the experiment under modest sample requirements (small volumes (~300 μl), low protein concentrations (~2 mg/ml) of relatively small molecules (GFPssrA, ~28 kDa)) and yet reach short exposure times (30 seconds) and obtain structural information during the active unfolding process.

Since SANS intensities are proportional to the product of sample concentration (in mg/ml) and molecular weight^19^, it is feasible to reduce exposure times further (to a few seconds) for larger substrates (>50 kDa) and for slightly higher concentrations (5–10 mg/ml). (In the case of the GroEL-GroES system (~1 MDa), *I*(0) intensities and radii of gyration have been reported with a time resolution of a few seconds by using a stopped-flow SANS device^28^). Thus, the approach can be systematically applied to a wide range of biological systems, in particular large molecular assemblies implying mechanical movements to ensure biological functions, including transcription processes, molecular motors, protein degradation (unfoldases and proteases) and refolding and quality control systems (chaperones). While being well above the time resolution (~100 ps) of pump-probe SAXS experiments at last-generation synchrotrons^29^ SANS does not induce radiation damage and has the advantage to distinguish between the macromolecular machine and its substrate within the assembled, active complex by using (per)deuteration of individual partners in combination with contrast variation (H_2_O:D_2_O solvent ratio)^21^. Finally, it is important to mention that the use of an archaeal, thermo-activatable system offered many technical advantages allowing a better control for the activity of the PAN complex in this biophysical study, in particular it was possible to slow down the unfolding reaction by working at temperatures below the physiological optimum.

### PAN destabilizes and releases aggregate-prone GFPssrA substrates within minutes

The time-resolved SANS study, coupled with online fluorescence spectroscopy, shows that native, monomeric GFP starts to disappear in the presence of PAN and ATP within 1–2 min under the experimental conditions applied (Figs 2 and 3). Of particular importance was the finding that the average model, 45 s after the beginning of the unfolding reaction (Fig. 2, red), displayed the same volume as native GFP (Fig. 2, grey) but with a more elongated shape (15–20 Å increase in D_max_). This shape therefore represents an average model for a population of native GFP molecules and unfolded intermediates and is compatible with a directional unfolding process, probably starting from the C-terminal side which is recognized *via* the ssrA tag by the N-terminal side of PAN. Even though a minor contribution from first small aggregates cannot be ruled out, it is interesting to note that the observed increase of D_max_ is in good agreement with recent results from single-molecule force spectroscopy on ClpXP^26^,^30^ which found an average length of the smallest substrate translocation steps of approximately five amino acids (i.e. ~18 Å assuming a peptide group dimension of 3.6 Å).

Shortly after this first elongation/destabilization step, and concomitantly to a loss of fluorescence, GFP aggregates appear in solution as indicated by the subsequent increase of the scattered intensity in the forward direction *I*(0) (Supplementary Table S1) and the generated *ab initio* models which display elongated shapes with increasing volumes over time as an indicator of the size distribution of the aggregates (Fig. 2). The disappearance of fluorescence toward the end of the process (~50 min, Fig. 3) implies total quenching of the fluorophore embedded into the β barrel of the folded GFP. Both the disappearance of the population of natively folded GFP as well as the decrease of fluorescence could be adequately described by single, exponential decay functions with specific characteristic time constants (Eq. 2, Supplementary Eq. 4, Fig. 3). Interestingly, the half-life of native, monomeric GFP found here (~1 min 45 s) is comparable to the time based on biochemical assays of ATP consumption rates of different ATPase systems who found that several hundred ATP molecules are needed to unfold one GFPssrA at hydrolyses rates of a few ATP per second^10^,^31^.

**Figure 6:**
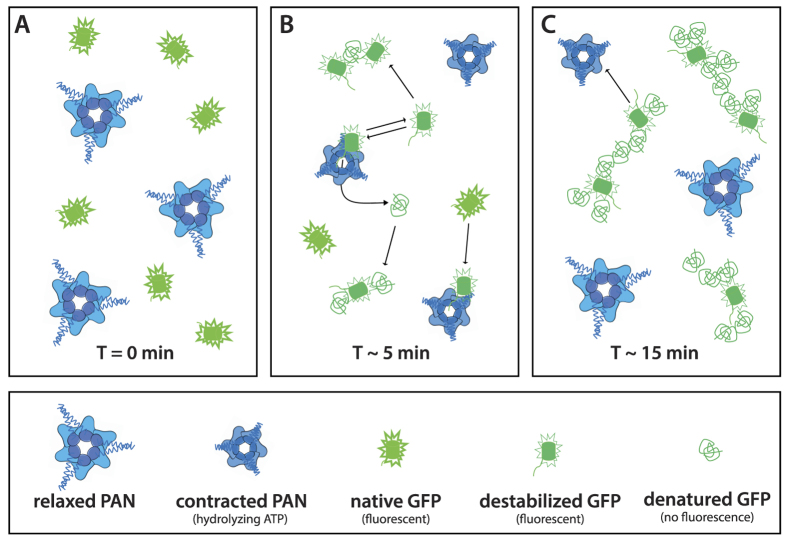
Putative scheme representing GFP unfolding by PAN. (**A**) At the beginning of the reaction PAN is in a relaxed, extended state and all GFP molecules are in their native state with maximum fluorescence. (**B**) After a few minutes PAN reaches its maximum activity and destabilizes/unfolds GFP molecules. GFP molecules are either released in a destabilized, yet fluorescent state or in a completely denatured, unfluorescent state and form first, small aggregates. (**C**) At a later state all natively folded GFP molecules have disappeared and all destabilized or denatured GFP molecules are clustered within aggregates. Occasionally, PAN interacts with clusters and denatures the destabilized members.

Surprisingly, while the loss of fluorescence followed equally an exponential decay, it occurred at a significantly slower rate with a characteristic half-life of 7 min (Fig. 3, blue curve). The discrepancy between the half-lives of native, monomeric GFP structures and the loss of fluorescence supports a “trial-and-error” mechanism of unfolding, proposed for several AAA+ protease systems and which states that several unsuccessful unfolding attempts with high ATP consumption are in general engaged prior to terminal, successful substrate unfolding^26^,^32^. In the experimental conditions in this work, unsuccessful unfolding attempts would produce a population of (locally) destabilized GFP proteins, putatively exposing hydrophobic patches, and thus promoting aggregation, but that would initially still be able to fluoresce (Fig. 6). As a consequence, the population of isolated, natively folded proteins would decrease quickly while the loss of fluorescence would be slower.

Due to the absence of the 20S catalytic particle in the reaction volume, it can be assumed that unfolded or destabilized GFPssrA were released in solution without being degraded into small peptides. Interestingly, the continuously growing tubular forms with a constant diameter corresponding roughly to the dimension of native GFP molecules (Fig. 2) suggest that PAN-unfolded (or destabilized) GFP, in the absence of associated 20S particles, spontaneously refolds into some “molten globule” form and aggregates into a “pearl necklace” arrangement (Fig. 6B,C), probably interacting *via* exposed hydrophobic patches. It is noteworthy to stress that the GFP aggregates due to unfolding by PAN are slightly different from the ones appearing after forced thermal denaturation (Supplementary Fig. S5), thus suggesting differences in the two molecular unfolding and aggregation mechanisms. The swift appearance of GFP aggregates within 1–2 min, provoked by PAN *in vitro* in the absence of a proteolytic partner, strongly underlines the need for an intimate and tightly regulated cooperation/association between AAA+ unfoldases and their proteolytic partners, and/or chaperons or other co-factors *in vivo* in order to avoid uncontrolled accumulation of potentially harmful or toxic protein aggregates^33^.

### A reversible, nucleotide-induced, large contraction of PAN accompanies its substrate unfolding activity

The time-resolved SANS data reveal an ATP-induced, reversible contraction of the *Mj*PAN complex in solution (~10% diminution of its radius of gyration *R*_G_ and maximum dimension D_max_) during substrate unfolding on the time scale of minutes (Figs 4 and 5, Supplementary Table S1). The relative amplitude of contraction observed here is slightly larger than the one reported by SAXS from p97 which displayed a 5–9% reduction of *R*_G_ of the apo-state upon binding of several non-hydrolysable ATP analogues, but in the absence of substrate^34^.

In atomic resolution structures of AAA+ proteases available so far, only rotations of the ATPase rings or movements of the pore loops have been described as responses to nucleotide binding and hydrolysis^17^. Indeed, a comparison of the theoretical SANS curves of nucleotide-free (PDB ID 3HTE) with nucleotide-bound (PDB ID 3HTE) ClpX bacterial AAA+ unfoldase with CRYSON^35^ predicts a contraction of the radius of gyration *R*_G_ from 43 to 42 Å, i.e. a relative decrease of only 2–3%. If one assumes a similar overall topology for the C-terminal parts of both systems the much higher relative decrease of *R*_G_ observed here for PAN suggests a more pronounced relative displacement of the centres of mass of its six subunits upon nucleotide binding than the motions detected in ClpX. Furthermore, the parallel axis theorem^36^,^37^ predicts that a rotation of a rigid subunit around its centre of mass does not change the overall radius of gyration of the complex it forms with other rigid partners. Therefore, a 10% reduction of the *R*_G_ implies a significant translational displacement of subunit centres toward the centre of the PAN complex, possibly combined with a conformational contraction of individual subunits and involving motions of the N-terminal coiled-coil domains (Fig. 4) as predicted by MD simulations^38^.

Importantly, since hydrogenated GFP molecules were well-matched at 42% D_2_O (Supplementary Fig. S2) it can be excluded that the changes in *R*_G_ were due to aggregated GFP molecules. Likewise, the relatively constant values of *I*(0) intensities (Supplementary Table S1) and the values of *R*_G_, compared to the ones of the SEC-SAXS experiment (Supplementary Fig. S1) preclude a significant presence of oligomeric PAN forms other than the hexamer. Unfortunately, in the absence of atomic resolution models of *Mj*PAN, the SANS data did not allow us to determine if the contraction is due to a symmetric movement of the six subunits or to an asymmetric movement concerning only some of the subunits and depending on the number of bound nucleotides (fully/partially loaded nucleotide states) as suggested recently^10^,^39^,^40^,^41^.

Interestingly, it was possible to describe the reversible PAN contraction by a simple bi-exponential law for the radius of gyration vs. time (Eq. 3 and Fig. 5), suggesting a two-state model comprising an open, relaxed state, and a closed, contracted state (Fig. 4). Both PAN contraction and relaxation occurred on a time scale of a few hundred seconds, and after relaxation PAN reached again its initial conformation indicating that the conformational switch, fuelled by ATP hydrolysis, is reversible and needed as a main mechanical force to allow cyclic substrate unfolding. The slight asymmetry of both times could be due to differences in ATP catalysis and ADP off-rates^10^ and/or to a smaller population of active, contracted PAN as GFP substrates diminish with time.

## Methods

### Protein production and biophysical characterization

*Methanocaldococcus jannaschii (Mj*PAN) and ssrA-tagged Green Fluorescent Protein (GFPssrA) were expressed in *Escherichia coli* in hydrogenated and perdeuterated state and purified according to standard procedures (see details in Supplementary Material and Methods).

Protein samples were characterized by negative-stain electron microscopy, mass spectrometry and small angle X-ray scattering, coupled to online size exclusion chromatography (SEC-SAXS) at the BioSAXS beamline BM29 at ESRF (see Supplementary Material and Methods).

*Mj*PAN interaction with GFPssrA was characterized by surface plasmon resonance (details in Supplementay Material and Methods).

### Time-resolved SANS data collection and analysis

SANS data sets were recorded on the D22 diffractometer at the Institut Laue Langevin (ILL, Grenoble, France), coupled to a specifically developed online fluorescence device (see details in Supplementary Material and Methods). 300 μl of the reaction mixtures of deuterated PAN (dPAN) with hydrogenated GFPssrA (hGFPssrA) as well as the inverse labelling scheme (Supplementary Tables S2 and S3) were prepared and pipetted in the quartz cells a few seconds before putting them into a thermo-stated sample rack (Supplementary Fig. S7). SANS datasets were recorded continuously in 30 second frames, in parallel to fluorescence data.

One-dimensional scattering intensities *I*(*q*) (in cm^−1^) were obtained from two-dimensional images using standard ILL software^42^. (*q* = (4*π/λ)*sin*θ*, with *λ* being the neutron wavelength and 2*θ* the scattering angle). Buffers were subtracted from the respective samples with the software PRIMUS^43^. The radii of gyration, *R*_G,_ and forward scattered intensities, *I*(0), were extracted using the Guinier approximation^44^. Theoretical *I*(0) values (in cm^−1^) were used for calibration of the molecular masses using the following relationship:


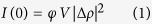


where *φ* is the volume fraction and *V* the solvent-excluded volume (in cm^3^) of the macromolecules in solution and Δ*ρ* the contrast (in cm^−2^) between the macromolecule and the solvent.

The program OLIGOMER^43^ was used to fit populations of natively folded GFPssrA, *P*_nat_(*t*), and the aggregates formed, *P*_agg_(*t*). The time dependence of the disappearance of the population of natively folded GFP was fitted by a single exponential decay function with a characteristic time constant τ:


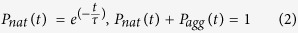


The time dependence of the PAN radius of gyration during substrate processing (from the dPAN:hGFPssrA data in 42% D_2_O) was fitted by a two-exponential function


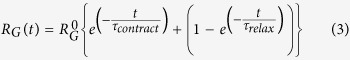


τ_contract_ and τ_relax_ are the characteristic time constants, describing the contraction and the relaxation, respectively, of the PAN complex during substrate unfolding, and 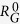 is the relaxed (i.e. the initial and final) radius of gyration.

Full details on SANS data analysis are available in the Supplementary Material and Methods.

## Additional Information

**How to cite this article**: Ibrahim, Z. *et al*. Time-resolved neutron scattering provides new insight into protein substrate processing by a AAA+ unfoldase. *Sci. Rep.* **7**, 40948; doi: 10.1038/srep40948 (2017).

**Publisher's note:** Springer Nature remains neutral with regard to jurisdictional claims in published maps and institutional affiliations.

## Supplementary Material

Supplementary Information
