# Supplementary material for: Time-resolved neutron scattering provides new insight into protein substrate processing by a AAA+ unfoldase
Source: Sci Rep. 2017 Jan 19;7:40948. doi: 10.1038/srep40948 (PMC5244417; doi:10.1038/srep40948)
Supplement: Supplementary Information [file srep40948-s1.pdf]

Supplementary Information

Time-resolved neutron scattering provides new insight into protein substrate processing by a AAA+ unfoldase

Ziad Ibrahim[†,§,‡,#], Anne Martel[#], Martine Moulin[#], Henry S. Kim[†,§,‡], Michael Härtlein[#], Bruno Franzetti[†,§,‡], Frank Gabel[†,§,‡,#,*]

[†]Université Grenoble Alpes, Institut de Biologie Structurale, 38044 Grenoble, France

[§]Centre National de la Recherche Scientifique, Institut de Biologie Structurale, 38044 Grenoble, France

[‡]Centre à l'Energie Atomique et aux Energies Alternatives, Institut de Biologie Structurale, 38044 Grenoble, France

[#]Institut Laue-Langevin, 38042 Grenoble, France

Supplementary Figures

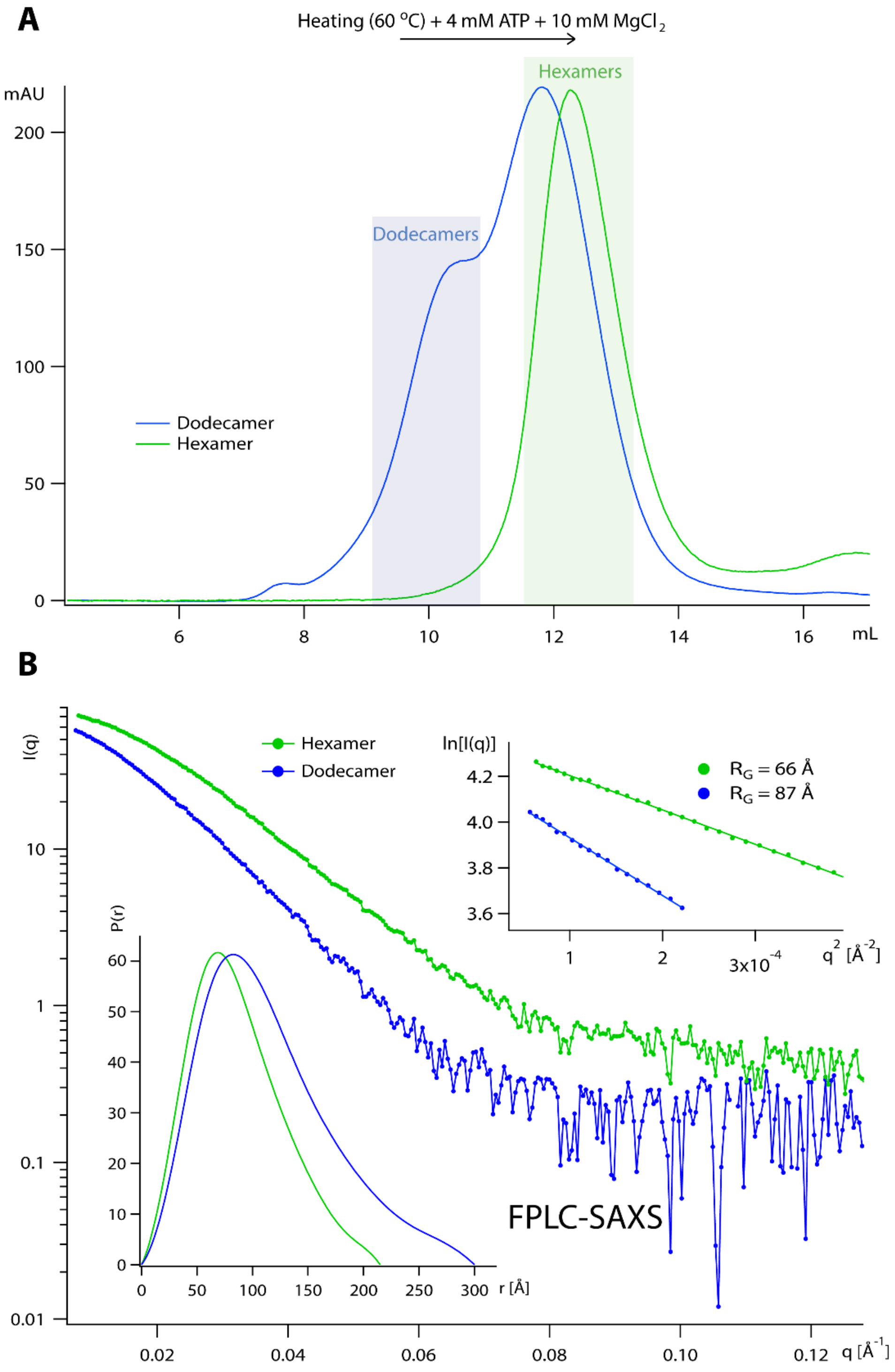

Figure S1: SEC-SAXS study of the *Mj*PAN complex in its dodecameric and hexameric states
(A) Superpose 6 column gel filtration profiles of dodecameric and hexameric *Mj*PAN. The shaded rectangles indicate the frame zones used for averaging and analysis of the SAXS curves corresponding to both oligomeric forms. Individual frames were collected with an exposure time of 4 seconds. (B) Scattering curves, pair distance distribution functions P(r) (bottom inset) and Guinier plots (top inset) from dodecameric (blue points) and hexameric (green points) *Mj*PAN, recorded on an FPLC coupled to the BioSAXS beamline BM29 (ESRF Grenoble). The hexameric form of the complex was generated by heating the dodecamers at 60 °C with 4 mM ATP and 10 mM $MgCl_2$ before re-injecting the protein solution on the Superose 6 column. Each scattering curve corresponds to the average of 10 frames collected from the shaded peak areas.

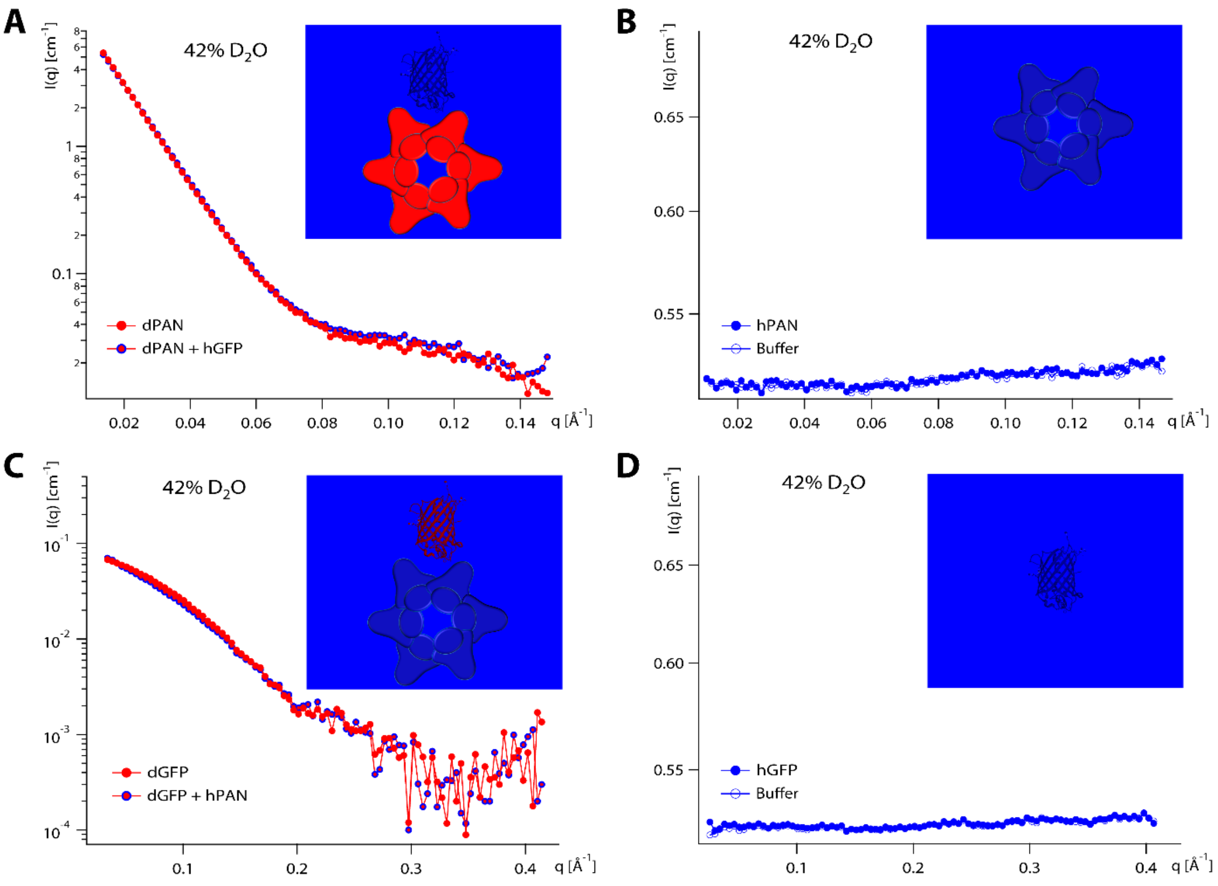

Figure S2: SANS control experiments on hydrogenated (h) and deuterated (d) proteins and their complexes validate the SANS strategy

(A) dPAN (5 mg/ml) in the presence (blue) or absence (red) of hGFPssrA (1 mg/ml). (B) hPAN (2 mg/ml) (full circles) superimposed with its corresponding buffer (open circles). (C) dGFPssrA (2 mg/ml) in the presence (blue) or absence (red) of hPAN (10 mg/ml). (D) hGFPssrA (4 mg/ml) (filled blue circles) superimposed with its corresponding buffer (open blue circles). All SANS data were recorded at 42% D_2O and the very good superposition of the respective curves over the total *q* range shows a negligible contribution of the hydrogenated proteins to the final signal. All curves were measured at room temperature in a static mode and without ATP in a buffer containing 20 mM Tris/HCl pH 7.5, 100 mM NaCl and 10 mM $MgCl_2$.

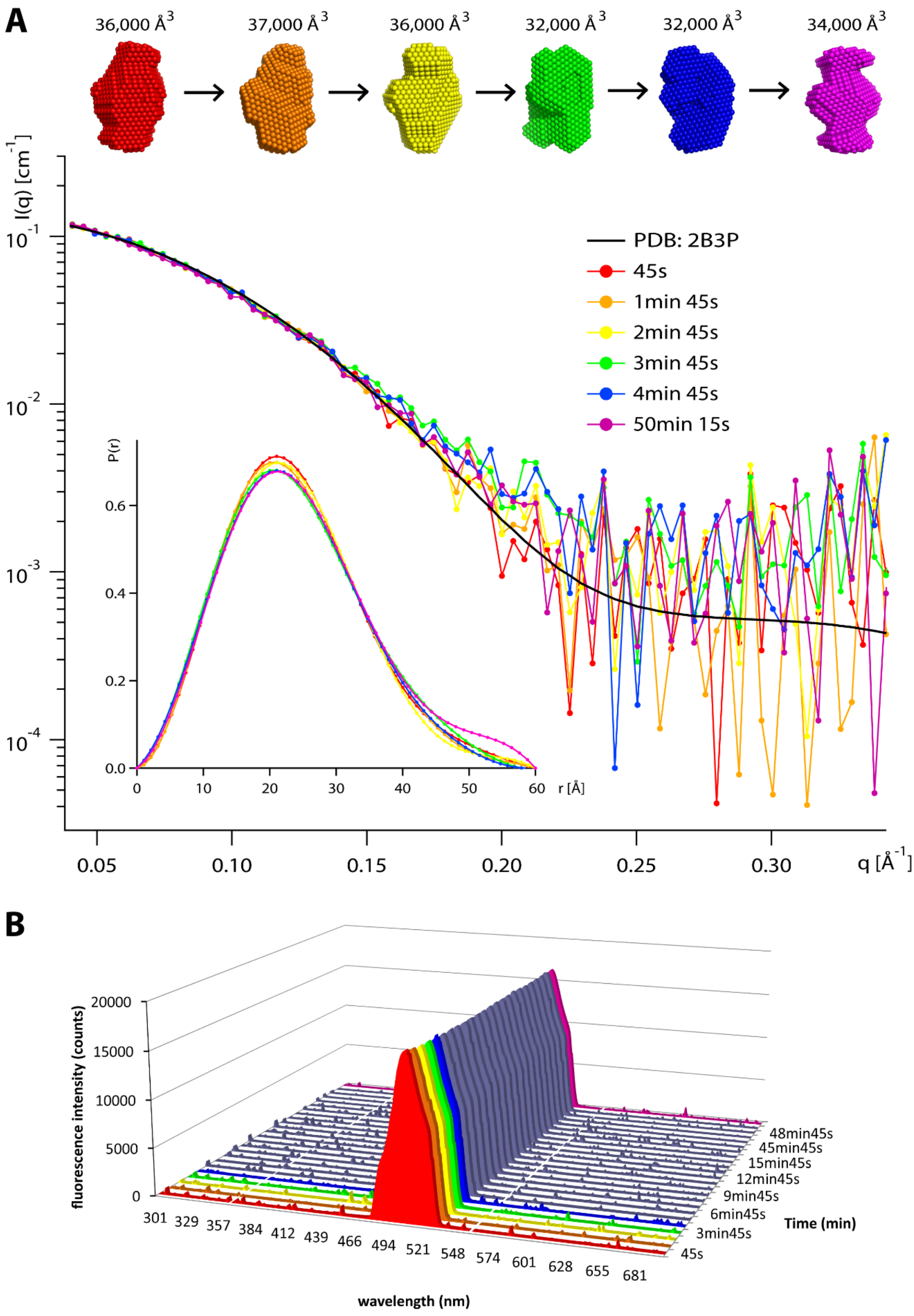

Figure S3: structural stability of isolated GFPssrA at 55 °C in solution
(A) SANS curves of dGFPssrA (2 mg/ml) in the absence of hPAN recorded at 55 °C with 30 second exposure time per curve. The scattering profiles were stable over time showing no thermal unfolding of isolated GFP. *Ab initio* envelopes of dGFPssrA, volumes calculated from each model and pair distance distribution functions P(r) (GNOM [1]) are equally shown. (B) UV fluorescence spectroscopy emission spectra of dGFPssrA in the absence of hPAN, measured on the same sample and at the same time as the SANS measurements show that the GFP fluorescence signal remained stable over time (same color code as in (A)).

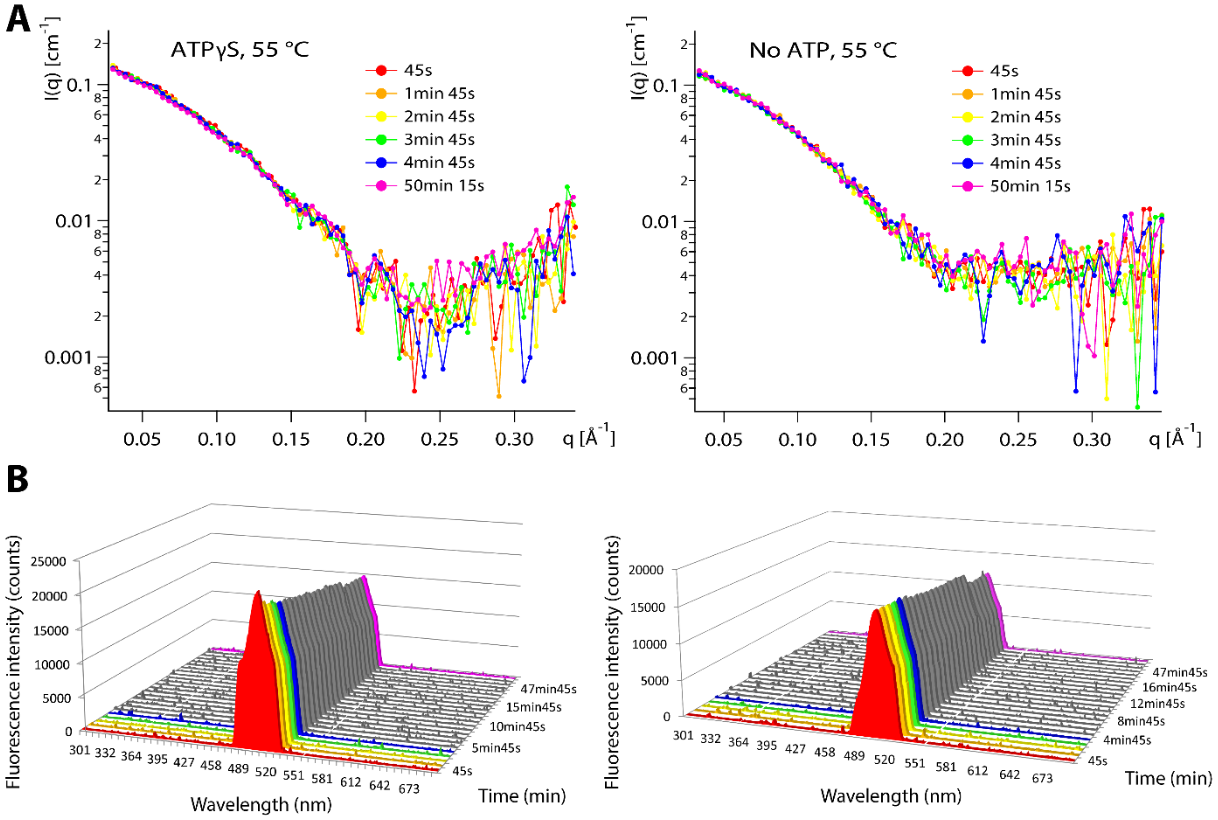

Figure S4: ATPγS or the absence of nucleotide prevent GFPssrA unfolding by PAN
(A) SANS curves recorded on dGFPssrA (2 mg/ml) in the presence of hPAN (10 mg/ml) without nucleotide (left) or with ATPγS (right) at 55 °C during the first 5 and after 50 minutes with 30 s exposure time per curve. The scattering profiles were stable over time showing no unfolding of GFP under these conditions. (B) UV fluorescence spectroscopy emission spectrums of GFPssrA in the presence of PAN without nucleotide (left) or with ATPγS (right), recorded during 50 minutes of the unfolding reaction on the same samples and in parallel to the SANS measurements (same color code as in (A)). Please note the discontinuity of the time scale in the fluorescence spectra which results in an intensity step after 15min.

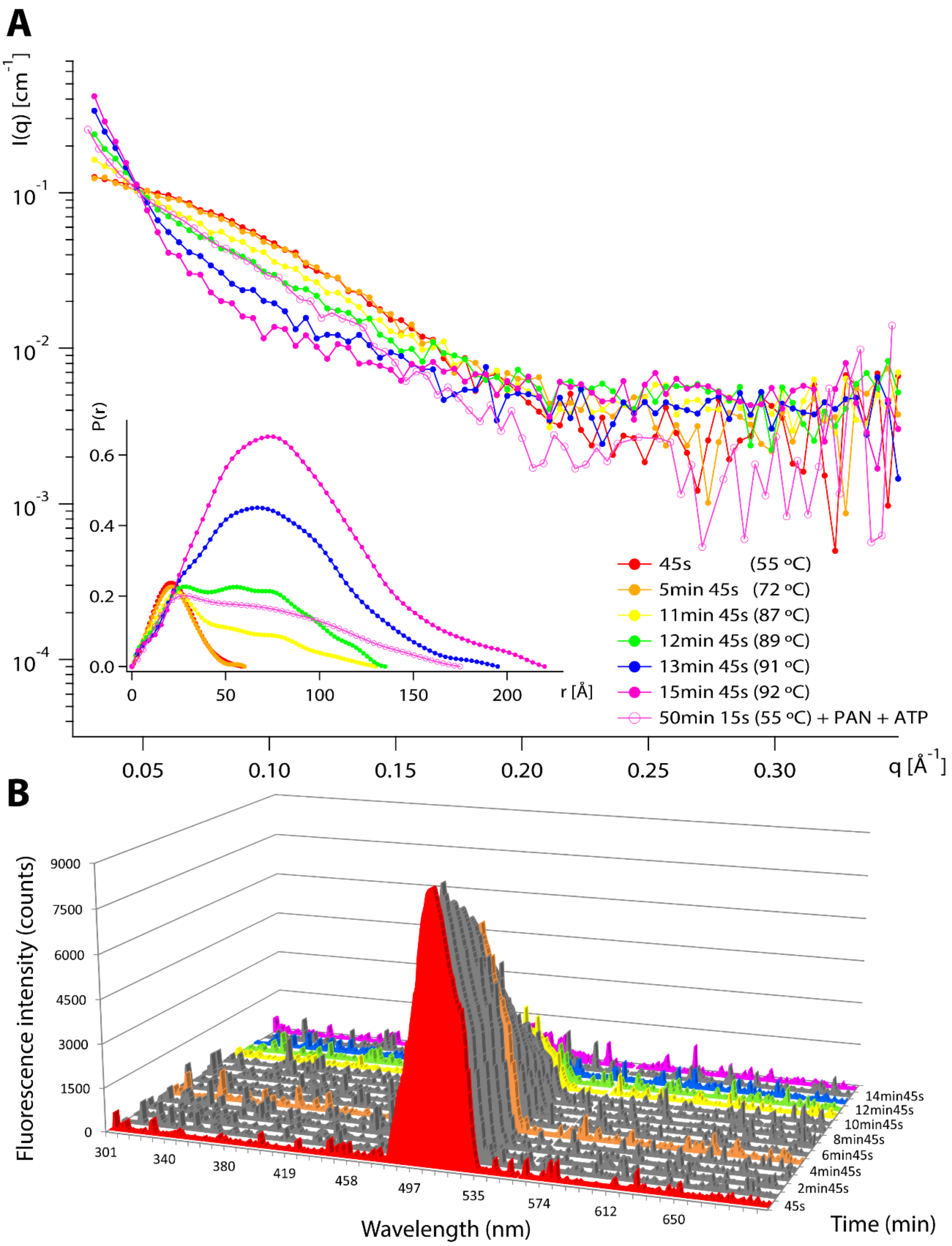

Figure S5: Forced thermal denaturation and aggregation of GFPssrA

(A) SANS data recorded on isolated dGFPssrA (2 mg/ml) at different times during a temperature ramp from 55 to 95 °C and dGFPssrA data in the presence of PAN (invisible) and ATP after 50 min 15 s at 55 °C. The scattering profiles show a pronounced evolution over time indicating that GFP is being unfolded and aggregates in solution. (B) UV fluorescence spectroscopy emission spectra of dGFPssrA measured concomitantly of the thermal unfolding process on the same sample and at the same time as the SANS measurements show a clear decrease of the GFP fluorescence signal (same color code as in (A)).

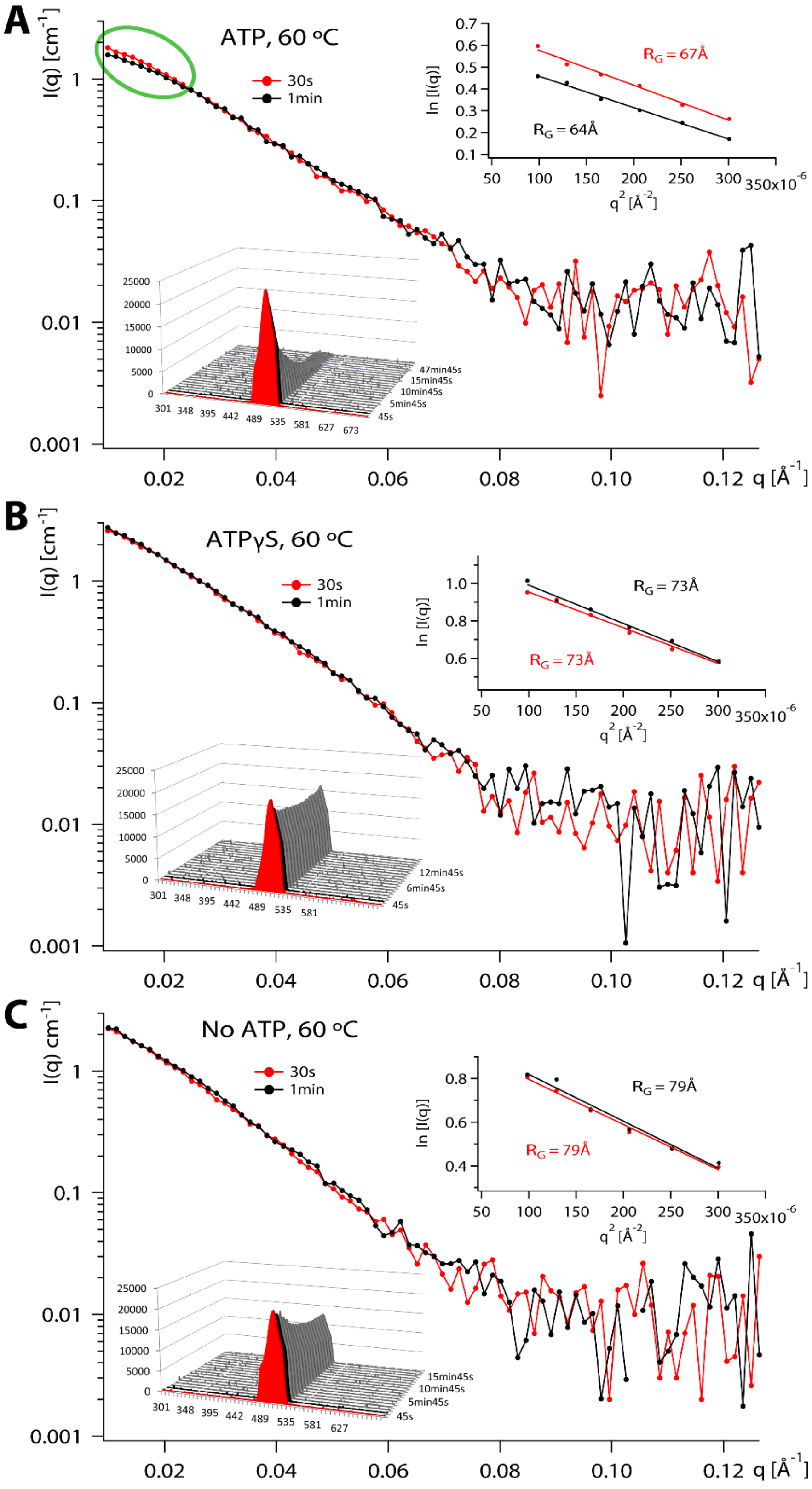

Figure S6: Absence of conformational changes in the absence of hydrolysable ATP

SANS curves recorded at 45 s and 1 min 15 s on dPAN (3 mg/ml) in the presence of hGFPssrA (4.8 mg/ml) at 60 °C in the presence of ATP (A), ATPγS (B) or without nucleotide (C) with 30 s exposure time per curve. Insets: Guinier plots and UV fluorescence spectroscopy emission spectra of GFP in each condition recorded during the first 15 min of the unfolding reaction on the same samples and at the same time as the SANS measurements. In the absence of ATP or in the presence of ATPγS, the fluorescence signal remained relatively stable over time with a very small decrease due to the interaction with PAN. Please note that the samples in plots (B) and (C), due to the absence of ATP and in contrast to plot (A), contained a small fraction of PAN dodecamers (as checked by gel filtration) and the radii of gyration were therefore increased.

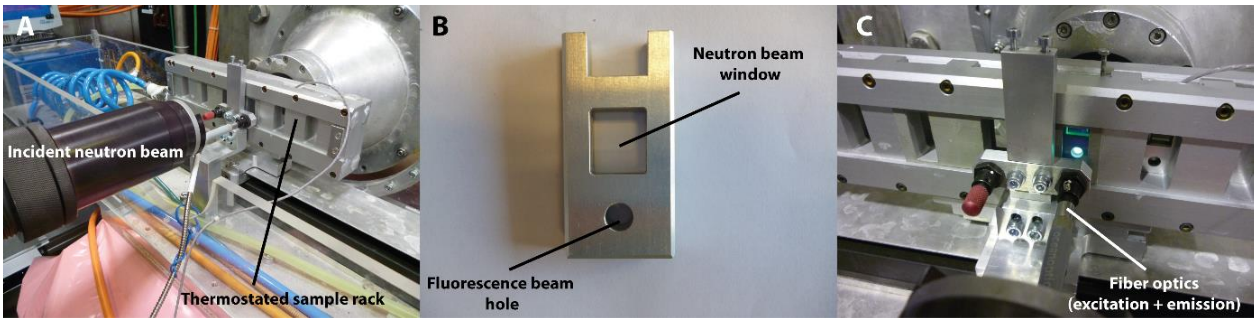

Figure S7: SANS-fluorimeter setup developed and mounted on the D22 beamline at the Institut Laue Langevin (ILL, Grenoble, France)
A) Incoming neutron beam and thermostated rack. B) Aluminum cell holder featuring the neutron and fluorescence windows that accommodates the quartz cell with PAN-GFP solutions. C) Excitation and emission fluorescence fibers with fluorescing GFP solution.

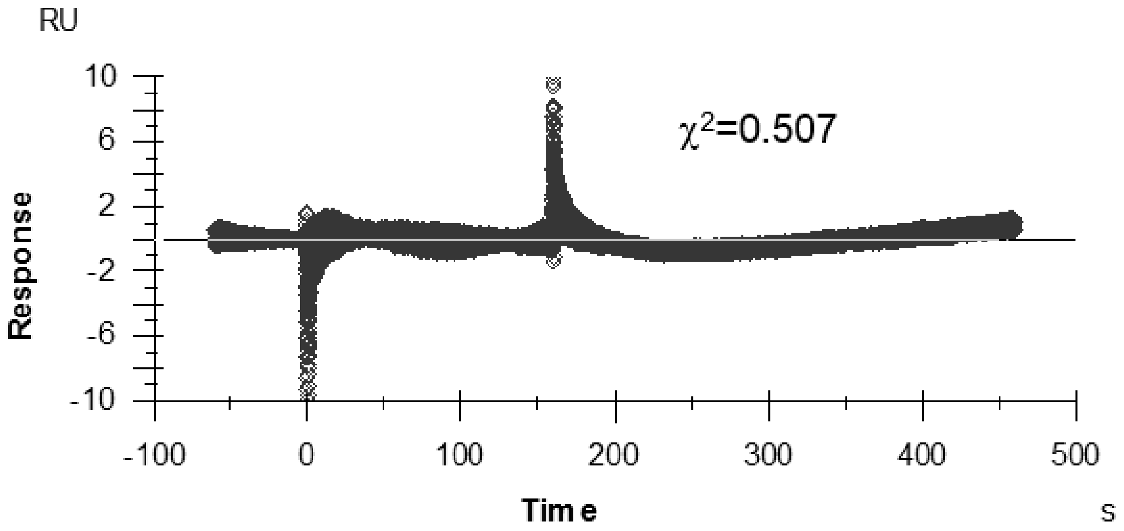

Figure S8: Residual plot and corresponding χ^2 value for the fit of 1:1 binding model [2] to the PAN-GFPssrA SPR data presented in Fig. 1B.

	R_G [Å]	D_{max} [Å]	$I(0)$ [cm^{-1}]
dGFP/hPAN (ATP, 55°C)			
45s	19.8 ± 0.5	75 ± 5	0.16 ± 0.01
1min 45s	24.5 ± 0.9	110 ± 5	0.19 ± 0.01
2min 45s	31.2 ± 0.8	125 ± 5	0.25 ± 0.01
3min 45s	35.1 ± 0.7	145 ± 5	0.31 ± 0.01
4min 45s	37.6 ± 0.6	155 ± 5	0.35 ± 0.02
50min 15s	41.1 ± 0.6	175 ± 5	0.38 ± 0.02
dGFP (55°C)			
45s	18.0 ± 1.0	60 ± 5	0.14 ± 0.01
1min 45s	17.5 ± 1.0	60 ± 5	0.14 ± 0.01
2min 45s	17.9 ± 0.9	60 ± 5	0.14 ± 0.01
3min 45s	17.5 ± 1.0	60 ± 5	0.14 ± 0.01
4min 45s	17.4 ± 1.0	60 ± 5	0.14 ± 0.01
50min 15s	19.5 ± 0.8	60 ± 5	0.15 ± 0.01
dGFP/hPAN (no ATP, 55°C)			
45s	19.2 ± 0.7	60 ± 5	0.14 ± 0.01
1min 45s	18.6 ± 0.7	60 ± 5	0.13 ± 0.01
2min 45s	20.6 ± 0.6	60 ± 5	0.14 ± 0.01
3min 45s	18.5 ± 0.7	60 ± 5	0.13 ± 0.01
4min 45s	20.2 ± 0.6	60 ± 5	0.14 ± 0.01
50min 15s	20.4 ± 0.6	60 ± 5	0.14 ± 0.01
dGFP/hPAN (ATPγS,55°C)			
45s	18.6 ± 0.6	60 ± 5	0.14 ± 0.01
1min 45s	18.8 ± 0.7	60 ± 5	0.14 ± 0.01
2min 45s	18.7 ± 0.8	60 ± 5	0.14 ± 0.01
3min 45s	19.3 ± 0.7	60 ± 5	0.14 ± 0.01
4min 45s	18.6 ± 0.7	60 ± 5	0.14 ± 0.01
50min 15s	20.4 ± 0.7	60 ± 5	0.14 ± 0.01
dGFP (thermal unfolding)			
45s (55°C)	18.4 ± 1.0	60 ± 5	0.14 ± 0.01
5min 45s (72°C)	19.4 ± 0.9	60 ± 5	0.14 ± 0.01
11min 45s (87°C)	30.2 ± 0.6	125 ± 5	0.21 ± 0.01
12min 45s (89°C)	43.6 ± 0.5	135 ± 5	0.44 ± 0.02
13min 45s (91°C)	53.0 ± 0.4	165 ± 5	0.83 ± 0.03
15min 45s (92°C)	57.8 ± 0.3	165 ± 5	1.22 ± 0.04
dPAN/hGFP (ATP, 60°C)			
45s	67.6 ± 1.1	220 ± 5	1.69 ± 0.01
4min 45s	62.1 ± 1.6	190 ± 5	1.54 ± 0.01
50min 15s	67.3 ± 1.5	220 ± 5	1.73 ± 0.01
dPAN/hGFP (noATP, 60°C)			
45s	79.1 ± 1.6	240 ± 5	2.73 ± 0.01
1min 15s	79.1 ± 1.5	240 ± 5	2.78 ± 0.01
dPAN/hGFP (ATPγS, 60°C)			
45s	72.7 ± 1.0	235 ± 5	3.05 ± 0.01
1min 15s	73.5 ± 1.0	235 ± 5	3.13 ± 0.01

Table S1: SANS parameters of the GFPssrA and PAN calculated from data recorded during SANS experiments. The radii of gyration (R_G) and the $I(0)$ intensities are extracted from the Guinier plots. The maximum dimension (D_{max}) is extracted from the P(r) function by indirect Fourier transform using GNOM [1]. The theoretical values of $I(0)$ for GFP monomers and PAN hexamers using Eq. 1 were 0.12±0.03 and 2.0±0.5 cm^{-1}, respectively.

Protein/component (h: hydrogenated, d: deuterated)	Final concentration	Volume (initial concentration)	Buffer
hPAN	10 mg/ml (33.5 µM)	150 µl (20 mg/ml)	H_2O
dGFPssrA	2 mg/ml (72.3 µM)	60 µl (10 mg/ml)	D_2O
ATP/ATPγS	100 mM	66 µl (455 mM)	D_2O
$MgCl_2$	100 mM	12 µl (2500 mM)	H_2O
Buffer	/	12 µl	H_2O
Total	/	300 µl	42% D_2O

Table S2: Sample composition dGFPssrA-hPAN.
Buffer composition: Tris/HCl pH 7.5 (20 mM), $MgCl_2$ (10 mM), NaCl (100 mM).

Protein/component (h: hydrogenated, d: deuterated)	Final concentration	Volume (initial concentration)	Buffer
dPAN	3 mg/ml (10 µM)	90 µl (10 mg/ml)	H_2O
hGFPssrA	4.8 mg/ml (173.5 µM)	72 µl (20 mg/ml)	H_2O
ATP/ATPγS	100 mM	66 µl (455 mM)	D_2O
$MgCl_2$	100 mM	12 µl (2500 mM)	H_2O
Buffer	/	60 µl	D_2O
Total	/	300 µl	42% D_2O

Table S3: Sample composition dPAN-hGFPssrA.
Buffer composition: Tris/HCl pH 7.5 (20 mM), $MgCl_2$ (10 mM), NaCl (100 mM).

Supplementary Material and Methods

Recombinant protein cloning, expression and purification

The PAN gene from *Methanocaldococcus jannaschii* (*Mj*PAN), optimized for rare codons in *Escherichia coli*, was synthesized and cloned by *GeneCust Europe* (http://www.genecust.com) into a pET30a vector using NdeI/XhoI cloning sites to promote the expression of the protein of interest with a poly-histidine tag on its C-terminal side. *Mj*PAN was over-expressed in *E. coli* strain BL21 (DE3) at 37 °C during 4 h. The cells were re-suspended in 50 ml Tris-HCl 20 mM pH 7.5, NaCl 150 mM, Triton X-100 0.1% and Imidazole 5 mM and supplemented with 12.5 mg lysozyme (Euromedex), 2.5 mg DNase I grade II (Roche), 10 mg RNase (Roche), 50 mg Pefabloc SC (Roche) and 0.5 ml 2 M $MgSO_4$. Cells were disrupted by sonication with 10 cycles on/off of 30 s each at 4 °C. The lysate was clarified by centrifugation at 12,000 rpm for 1 h (JA20 rotor) and the supernatant was loaded onto a 5 ml Nickel column (HiTrap Chelating HP, GE Healthcare) equilibrated in 20 mM Tris/HCl pH 7.5, 10 mM $MgCl_2$, 100 mM NaCl and 5 mM Imidazole. After washing with the same buffer first with 20 mM and then with 50 mM imidazole, bound proteins were eluted with 20 ml at 500 mM imidazole.

After dialysis overnight against the same buffer without imidazole, the protein was purified by a 6 ml Resource Q anion exchange column (GE healthcare) equilibrated in 100 mM NaCl, 20 mM Tris-HCl, pH 7.5, 10 mM $MgCl_2$. After washing with the same buffer, bound proteins were eluted with a 20 CV (column volumes) linear salt gradient (0.10-0.35 M NaCl using the equilibration buffer). The fractions of the peak were combined and for further purification, the protein was loaded on a Superose 6 10/300 GL size exclusion column (GE healthcare) equilibrated in the buffer containing 100 mM NaCl, 20 mM Tris-HCl, pH 7.5, 10 mM $MgCl_2$. Protein absorbance at 280 nm was used to determine the concentration of purified protein samples (NanoDrop 2000 spectrophotometer, Thermo Scientific). The purified proteins were stored at –20 °C after the size-exclusion step. Before each use, PAN molecules were hexamerised and activated by heating at 60 °C for 30 min with 4 mM ATP and 10 mM $MgCl_2$ and the hexamers were re-injected on the Superose 6 10/300 GL size exclusion column as a last purification step and to monitor the complete hexamerisation of the molecules. The eluted volume corresponded to the hexameric form of the complex. The oligomerization state of PAN was also checked by SAXS and by negative stain electron microscopy.

The DNA sequence encoding the GFPssrA protein (containing an A206K mutation to minimize dimerization) was generated from synthetic DNA fragments and cloned by *GeneCust Europe*

(http://www.genecust.com) into the over-expression plasmid pET30a using NdeI/NotI cloning sites. GFPssrA is a variant of the green fluorescent protein, with its C-terminus fused to the 11-residue peptide ssrA (AANDENYALAA) [3]. In eubacteria this 11-mer targets proteins for degradation and the substrate specificity is usually determined through the placement of the degradation tags or degrons [4]. The resulting construct was used to transform *E. coli* strain BL21 (DE3) for the recombinant expression of the protein at 37 °C. GFPssrA was purified by ethanol extraction followed by Phenyl Sepharose 6 Fast Flow hydrophobic interaction chromatography as described [5] and resulted in a monomeric form of the protein as checked by gel filtration Superose 12 10/300 GL (GE Healthcare) and SANS.

Expression and purification of perdeuterated *Mj*PAN and GFPssrA proteins

Identical, engineered plasmids containing the genes of interest and encoding for both proteins, *Mj*PAN and GFPssrA, were used for the production of the hydrogenated and perdeuterated proteins. The perdeuterated proteins were expressed at the Institut Laue-Langevin Deuteration Laboratory (D-LAB) (Grenoble, France) in *E. coli* BL21 (DE3) bacterial strain. High-cell-density fermentation process in D_2O Enfors minimal medium [6] with d_8-glycerol (fully deuterated glycerol) as the carbon source and D_2O as solvent was used to grow bacteria at 30 °C to an OD_{600} of 11, followed by induction of protein expression by isopropyl-β-D-thio-galactoside (IPTG) to a final concentration of 1 mM. Bacteria were harvested when an OD_{600} of 16.6 was reached. The expressed perdeuterated proteins were purified using the same protocols as their hydrogenated counterparts. For purification of both hydrogenated and perdeuterated forms, H_2O-based buffer systems were used.

Negative-stain electron microscopy

After the size exclusion column step of the purification, 4 µl of the dodecameric and the hexameric fractions of *Mj*PAN sample (~ 0.1 mg/ml) were deposited onto carbon-coated 400-mesh copper grids. The samples were stained using 2% sodium silicotungstate pH 7.5 and air-dried. Images were taken under low-dose conditions in a T12 FEI electron microscope working at 120 kV and with a nominal magnification of 40,000 using an Orius SC1000 CCD camera.

Mass Spectrometry

LC/electrospray ionization-TOF-MS analysis was carried out on dPAN and dGFPssrA. The mass for dGFPssrA (28976.18 Da) gives an estimate of 69 % deuteration (without the exchangeable H) due to the H_2O based MS buffers and ~ 92 % deuteration combined with the

calculated mass (with deuteration of exchangeable H) in 100 % D_2O buffer. The mass of dPAN (52526.76 Da) gives an estimate of ~ 76 % deuteration (without the exchangeable H) and ~ 98% deuteration combined with the calculated mass (with deuteration of exchangeable H) in 100 % D_2O buffer. The Biological Scattering Tools website (http://psldc.isis.rl.ac.uk/) was used for the calculation of the number of exchangeable H in each protein.

Surface plasmon resonance (SPR)

Histidine-tagged PAN was purified as described above and diluted to a final concentration of 1 to 5 µM in HBS buffer (10 mM HEPES, pH 7.5, 150 mM NaCl). The protein was further diluted 10- to 50-fold in running buffer (10 mM HEPES, pH 7.5, 150 mM NaCl, 50 µM EDTA, 0.025 % surfactant P20; GE Healthcare) before being injected over the Ni-NTA coated sensor chip (Series S Sensor Chip NTA, GE Healthcare) surface at 10 µl/min at 45 °C to generate ~1500 response units (RU) of immobilized PAN. The analyte GFPssrA protein was serially diluted in running buffer at a range of concentrations indicated in Fig. 1B and injected at 45 °C (max. instrument temperature) at a flow rate of 50 µl/min for 3-4 min. Surface was regenerated after 5 min of dissociation by 1 min injection of 350 mM EDTA at 30 µl/min. Analyses of protein concentrations were done in duplicates and any background signal from a NTA-only reference flow cell was subtracted from every data set. Data were analyzed (Fig. S8) by a simple 1:1 Langmuir binding model [2] using the Biacore T200 evaluation software (Biaeval., GE Healthcare) to determine the kinetics (association and dissociation rate constants: k_a and k_d) as well as the affinity (K_D) of the PAN-GFPssrA interaction.

SEC-SAXS data collection

Size exclusion chromatography (SEC) small angle X-ray scattering (SAXS) experiments were carried out on the BM29 beamline at the European Synchrotron Radiation Facility (ESRF, Grenoble, France). To this end an on-line FPLC system (Viscotek GPCmax, Malvern Instruments) was attached directly to the sample-inlet valve of the beamline sample changer [7]. Protein samples were loaded into vials and automatically injected onto the column (Superose 6 10/300 GL, GE Healthcare) *via* an integrated syringe system. Buffers were degassed and a flow rate of 0.5 ml/min at 25 °C was used for all sample runs. The buffer was the same as described in the purification paragraph for the gel filtration column. Prior to each run, the column was equilibrated with 2 CV of buffer and the baseline was monitored. All data from the run were

collected at a wavelength λ = 0.99 Å using a sample-to-detector (PILATUS 1M, DECTRIS) distance of 2.81 m corresponding to a q-range of 0.008–0.45 $Å^{-1}$ where q is the momentum transfer ($q = \frac{4\pi}{\lambda}\sin\theta$) and 2θ the scattering angle. Approximately 550 frames with an exposure time of 4 seconds/frame were collected per sample run. Initial data processing was performed automatically using the EDNA pipeline [8], generating radially integrated, calibrated and normalized one-dimensional profiles for each frame. Initial frames were averaged to create the reference buffer and the frames collected from each elution peak (10 frames/peak), corresponding to the scattering of an individual purified species, were also averaged and subtracted from the reference buffer using the program PRIMUS [9]. Radii of gyration R_G and pair distance distribution functions P(r) were extracted as described in the "SANS data collection and analysis" section.

SANS data collection and analysis

SANS data sets were recorded on the D22 diffractometer (http://www.ill.eu/instruments-support/instruments-groups/instruments/d22) at the Institut Laue Langevin (ILL, Grenoble, France). For the static control experiments (match-points and masking of hydrogenated proteins, Fig. S2), 200 µl of the sample solutions were measured at room temperature in quartz cuvettes (Hellma 100QS) with 1 mm path length. Two configurations of the instrument were chosen, the sample was placed with a collimator/detector distances of 2/2 m for the GFPssrA and 5.6/5.6 m for *Mj*PAN. The wavelength was 6 Å in all cases.

For the time-resolved SANS experiments, 300 µl of the reaction mixture (Table S2 & S3) were prepared and pipetted in the quartz cells a few seconds before putting them in the thermo-stated sample rack already adjusted to the desired temperature needed for the enzymatic reaction (Fig. S7). The unfolding reaction immediately started once the cuvette was placed in the rack. The scattering curves were recorded with 30 s exposure times (plus an initial 15 seconds corresponding to the time delay and procedure to get out of the experimental hutch). In total, 180 scattering curves were recorded during a 90 min reaction time for each condition. However, since we did not observe changes after ~50 min, all data sets are displayed up to this time. Two main reaction mixtures were measured in a medium containing 42% D_2O allowing the matching of hydrogenated (h) proteins [10]: dGFPssrA:hPAN with a collimator/detector distance of 2.0/2.0 m and dPAN:hGFPssrA with a collimator/detector distance 5.6/5.6 m. Both kinetics were equally measured in different control experiments: with ATP, ATPγS or in the absence of nucleotide, at different temperatures (50, 55, 60 and 65 °C), at different PAN:GFPssrA

stoichiometry or in the absence of one of the protein partners. The corresponding buffers, the empty cell, the empty beam and boron/cadmium were measured in the same collimator/detector setups to perform data reduction (correction for detector efficiency, electronic noise, and sample holder scattering). Data reduction and radial integration over a two-dimensional image were performed using standard ILL software [11] and the data are presented in cm^{-1}.

The scattering curves of the buffers were subtracted from the respective sample scattering curves with the software PRIMUS [9]. The radii of gyration, R_G, and forward scattered intensities, $I(0)$, were extracted using the Guinier approximation, i.e. by a linear fit in an $\ln[I(q)]$ vs. q^2 plot [12]. Theoretical $I(0)$ values (in cm^{-1}) were used for absolute calibration of the molecular masses using the following relationship:

$$I(0) = \varphi V |\Delta\rho|^2 \qquad \text{(Eq. 1)}$$

where φ is the volume fraction and V the solvent-excluded volume (in cm^3) of the macromolecules in solution and $\Delta\rho$ the contrast (in cm^{-2}) between the macromolecule and the solvent. Volume fractions, solvent-excluded volumes and contrasts were calculated from literature [10] using the amino acid sequences (UniProtKB/Swiss-Prot entries P42212.1 and Q58576.1) and measured protein concentrations (2 and 3 mg/ml) of dGFPssrA and dPAN, respectively. The pair-wise distance distribution functions P(r) were generated with GNOM [1] by imposing P(r=0)=0 and P(r=D_{max})=0 (D_{max} = maximum distance found in the particle). The program DAMMIN [13] was used to generate low resolution envelopes of GFPssrA from the SANS data up to q_{max}= 0.3-0.35 $Å^{-1}$. As a quality control, the radii of gyration determined from the P(r) analyses were required to agree with the ones from the Guinier analyses. For the GFPssrA, the first experimental scattering curves recorded at the beginning of the unfolding were compared to the back-calculated scattering curves from the atomic model (PDB ID 2B3P) using the program CRYSON [14]. The program OLIGOMER [9] was used to fit the experimental scattering curves from the multicomponent mixture of the GFPssrA during the unfolding by PAN (native folded GFPssrA and GFPssrA aggregates) by the form factors of each component calculated by the program ffmaker (from OLIGOMER) to quantify their respective volume fractions in the mixture during different times of the unfolding. The time dependence of the disappearance of the population $P_{nat}(t)$ of natively folded GFP (dGFPssrA:hPAN data) and the appearance of an aggregated population $P_{agg}(t)$ was mathematically fitted by a single exponential decay functions with a characteristic time constant τ:

$$P_{nat}(t) = e^{(-\frac{t}{\tau})} \quad , P_{nat}(t) + P_{agg}(t) = 1 \qquad \text{(Eq. 2)}$$

The time dependence of the PAN radius of gyration during substrate processing (from the dPAN:hGFPssrA data) was fitted by a two-exponential function

$$R_G(t) = R_G^0 \left\{ e^{\left(-\frac{t}{\tau_{contract}}\right)} + \left(1 - e^{\left(-\frac{t}{\tau_{relax}}\right)}\right) \right\} \quad \text{(Eq. 3)}$$

$\tau_{contract}$ and τ_{relax} are the time relaxation constants of the PAN complex, describing its contraction and the relaxation, respectively, during substrate unfolding, and R_G^0 is the relaxed (i.e. the initial and final) radius of gyration. Both equations were fitted to the experimental data by the program IGOR Pro [15].

Online fluorescence measurement

In parallel to all kinetic SANS measurements, online fluorescence measurements were carried out. To this end, a special setup with an *in situ* spectrofluorimeter (Ocean Optics, 65000 pro) was implemented on the D22 beamline at the ILL (Grenoble, France) (Fig. S7). Fiber optics (IDIL Fibres Optiques) were connected to the SANS quartz cell containing 300 µl of the reaction volume and the excitation and emission wavelengths were filtered to be at 400 and 509 nm, respectively. The fluorescence emission spectrums (1 s) were recorded every 30 seconds and the fluorescence data were registered on the Nexus files [16], together with the SANS data. All spectrums were subsequently extracted and analyzed by the program HDFview (https://www.hdfgroup.org/products/java/hdfview/).

The decay of the initial fluorescence peak intensity I_{fluo} at 509 nm (normalized to 1) was fitted by a single exponential function with a characteristic time constant τ:

$$I_{fluo}(t) = e^{\left(-\frac{t}{\tau}\right)} \quad \text{(Eq. 4)}$$

Supplementary references

1. Svergun, D.I. Determination of the regularization parameter in indirect-transform methods using perceptual criteria. *J Appl Cryst* **25**, 495-503 (1992).
2. Day, E.S., Capili, A.D., Borysenko, C.W., Zafari, M. & Whitty, A. Determining the affinity and stoichiometry of interactions between unmodified proteins in solution using Biacore. *Anal Biochem* **440**, 96-107 (2013).
3. Weber-Ban, E.U., Reid, B.G., Miranker, A.D. & Horwich, A.L. Global unfolding of a substrate protein by the Hsp100 chaperone ClpA. *Nature* **401**, 90-93 (1999).
4. Benaroudj, N. & Goldberg, A.L. PAN, the proteasome-activating nucleotidase from archaebacteria, is a protein-unfolding molecular chaperone. *Nat Cell Biol* **2**, 833-839 (2000).
5. Samarkina, O.N. et al. Universal and rapid method for purification of GFP-like proteins by the ethanol extraction. *Protein Expression and Purification* **65**, 108–113 (2009).

6. Artero, J.-B., Härtlein, M., McSweeney, S. & Timmins, P. A comparison of refined X-ray structures of hydrogenated and perdeuterated rat gammaE-crystallin in H2O and D2O. *Acta Cryst* **61**, 1541-1549 (2005).
7. Round, A. et al. Determination of the GH3.12 protein conformation through HPLC-integrated SAXS measurements combined with X-ray crystallography. *Acta Crystallographica Section D* **69**, 2072-2080 (2013).
8. Incardona, M.-F. et al. EDNA: a framework for plugin-based applications applied to X-ray experiment online data analysis. *J Synchrotron Rad* **16**, 872-879 (2009).
9. Konarev, P.V., Volkov, V.V., Sokolova, A.V., Koch, M.H.J. & Svergun, D.I. PRIMUS: a Windows PC-based system for small-angle scattering data analysis. *J Appl Cryst* **36**, 1277-1282 (2003).
10. Jacrot, B. The study of biological structures by neutron scattering from solution. *Rep Prog Phys* **39**, 911-953 (1976).
11. Dewhurst, C. GRASP. *http://www.ill.fr/lss/grasp/grasp_main.html* (2002).
12. Guinier, A. Masson (1939).
13. Svergun, D.I. Restoring low resolution structure of biological macromolecules from solution scattering using simulated annealing. *Biophys J* **76**, 2879-2886 (1999).
14. Svergun, D.I. et al. Protein hydration in solution: Experimental observation by x-ray and neutron scattering. *Proceedings of the National Academy of Sciences of the United States of America* **95**, 2267-2272 (1998).
15. Gomez, J.F., Brioso, M.A., Machado, J.D., Sanchez, J.L. & Borges, R. New Approaches for Analysis of Amperometrical Recordings. *Annals of the New York Academy of Sciences* **971**, 647-654 (2002).
16. Maddison, D.R., Swofford, D.L. & Maddison, W.P. Nexus: An Extensible File Format for Systematic Information. *Systematic Biology* **46**, 590-621 (1997).
